# Antibody-induced dimerization of FGFR1 promotes receptor endocytosis independently of its kinase activity

**DOI:** 10.1038/s41598-017-07479-z

**Published:** 2017-08-02

**Authors:** Łukasz Opaliński, Aleksandra Sokołowska-Wędzina, Martyna Szczepara, Małgorzata Zakrzewska, Jacek Otlewski

**Affiliations:** Faculty of Biotechnology, Department of Protein Engineering, University of Wroclaw, Joliot-Curie 14a, 50-383 Wroclaw, Poland

## Abstract

Fibroblast growth factors (FGFs) and their plasma membrane-localized receptors (FGFRs) play a key role in the regulation of developmental processes and metabolism. Aberrant FGFR signaling is associated with the progression of serious metabolic diseases and human cancer. Binding of FGFs to FGFRs induces receptor dimerization and transphosphorylation of FGFR kinase domains that triggers activation of intracellular signaling pathways. Following activation, FGFRs undergo internalization and subsequent lysosomal degradation, which terminates transmission of signals. Although factors that regulate FGFR endocytosis are continuously discovered, little is known about the molecular mechanism that initiates the internalization of FGFRs. Here, we analyzed the internalization of antibody fragments in various formats that target FGFR1. We show that FGFR1-specific antibody fragments in the monovalent scFv format bind to FGFR1, but are not internalized into cells that overproduce FGFR1. In contrast, the same scFv proteins in the bivalent scFv-Fc format are efficiently internalized via FGFR1-mediated, clathrin and dynamin dependent endocytosis. Interestingly, the receptor tyrosine kinase activity is dispensable for endocytosis of scFv-Fc-FGFR1 complexes, suggesting that only dimerization of receptor is required to trigger endocytosis of FGFR1 complexes.

## Introduction

Fibroblast growth factor receptors (FGFRs) comprise a group of four receptor tyrosine kinases (RTKs) (FGFR1-FGFR4) that cooperate with extracellular fibroblast growth factors (FGFs) in the transduction of signals through the plasma membrane. The FGFRs-FGFs signaling cascades play fundamental role in organogenesis, angiogenesis, metabolism and tissue repair^1, 2^. The malfunction of the FGFRs-FGFs signaling axis leads to the developmental disorders and cancer^3–5^. FGFRs consist of an extracellular region that is composed of three Ig domains: D1, D2 and D3, from which D2 and D3 are involved in FGFs binding, a single transmembrane region and an intracellular protein kinase domain. Binding of FGFs to FGFRs stimulates receptor dimerization and induces conformational changes that lead to the activation of the receptor cytoplasmic tyrosine kinases. Activated FGFRs trigger signaling through the initiation of several pivotal pathways including PLCγ/PKC, Ras/Raf/ERK and PI3 kinase/PDK/Akt4.

Similarly to other RTKs, FGFRs undergo basal constitutive internalization from the plasma membrane. Ligand binding and subsequent FGFRs activation strongly enhances the internalization of FGFRs^6, 7^. FGFRs undergo either clathrin mediated endocytosis (CME) or clathrin independent endocytosis (CIE), depending on the FGFR type^7–9^. In the CME, RTKs are sequestered by clathrin lattices and separated from the plasma membrane by large GTPase dynamin^10^. CIE requires either remodeling of the actin cytoskeleton and membrane ruffling or caveolae^11^. Recent studies have led to the discovery of various factors that regulate endocytosis of FGFRs^12–18^. Following internalization, FGF-FGFR complexes are targeted to lysosomes for degradation or recycled to the plasma membrane^8^. The intracellular sorting of endocytosed FGFR-FGF complexes may be regulated by receptor ubiquitination^19^. Interestingly, in some cases internalized FGF-FGFR can escape lysosomal degradation via translocation through the endosomal membrane to the cytosol and nucleus^1^. It is widely accepted that the ligand-induced internalization of FGFRs serves as a negative regulator of receptor signaling at the plasma membrane^20^. Internalized RTKs can regulate signaling pathways also at the level of the endosomal membrane, indicating the role of endocytosis in the modulation of signal transduction^21^.

Despite the continuous progress in the understanding of the FGFRs trafficking, it is still largely unknown what actually triggers the internalization of FGFR1. Internalization of FGF1-FGFR1 complexes and subsequent translocation of FGF1 to the cytosol and the nucleus may occur independently of receptor kinase activity^22^. Internalization of model RTK, epidermal growth factor receptor (EGFR), is largely driven by the dimerization of EGFR and not by activation of receptor kinase^23, 24^. It was proposed that dimerization of EGFR brings together two sets of “endocytic codes” present in the cytoplasmic regions of EGFR that are recognized by cellular endocytic machineries^25^. Interestingly, not only natural ligands of RTKs can induce internalization of RTKs^26, 27^. Certain monoclonal antibodies against RTKs can activate receptors, inducing their endocytosis and subsequent degradation^28–30^.

Given the importance of FGFR-dependent signaling pathways in the development of numerous pathologies, various therapeutic strategies are designed to target FGFR signaling circuits^2^. One of the most promising approaches for selective treatment of FGFR overproducing cancers is based on the application of highly specific antibodies or antibody fragments fused with potent cytotoxic drugs that together form antibody drug conjugates (ADCs)^31–33^. In ADCs, antibodies confer specificity and facilitate internalization, as it occurs mainly via receptor mediated endocytosis. Interestingly, not all antibodies directed against cell surface receptors undergo efficient internalization and the molecular basis of this phenomenon is largely unknown^34, 35^. Since numerous antibodies against RTKs are either developed or already clinically used to treat RTKs-dependent cancers, it is of great importance to understand mechanism of antibody-induced internalization of RTKs.

Here, we studied the internalization of antibody fragments that target FGFR1. We show that antibody fragments in the bivalent format are efficiently internalized into the cells via receptor-mediated endocytosis. The internalization of scFv-Fc, similarly to the internalization of natural receptor ligand FGF1, requires clathrin and dynamin. Our data reveal that non-ligand induced dimerization of FGFR1 can trigger receptor endocytosis independently of receptor activation. This report provides novel insights into the biology of FGFR1 and may contribute to the design of highly internalizing, receptor non-activating antibodies that could be adequate for ADCs dedicated for treatment of FGFR1 overproducing cancers.

## Results

### FGFR1-specific antibody fragments do not interfere with the receptor function

**Figure 1:**
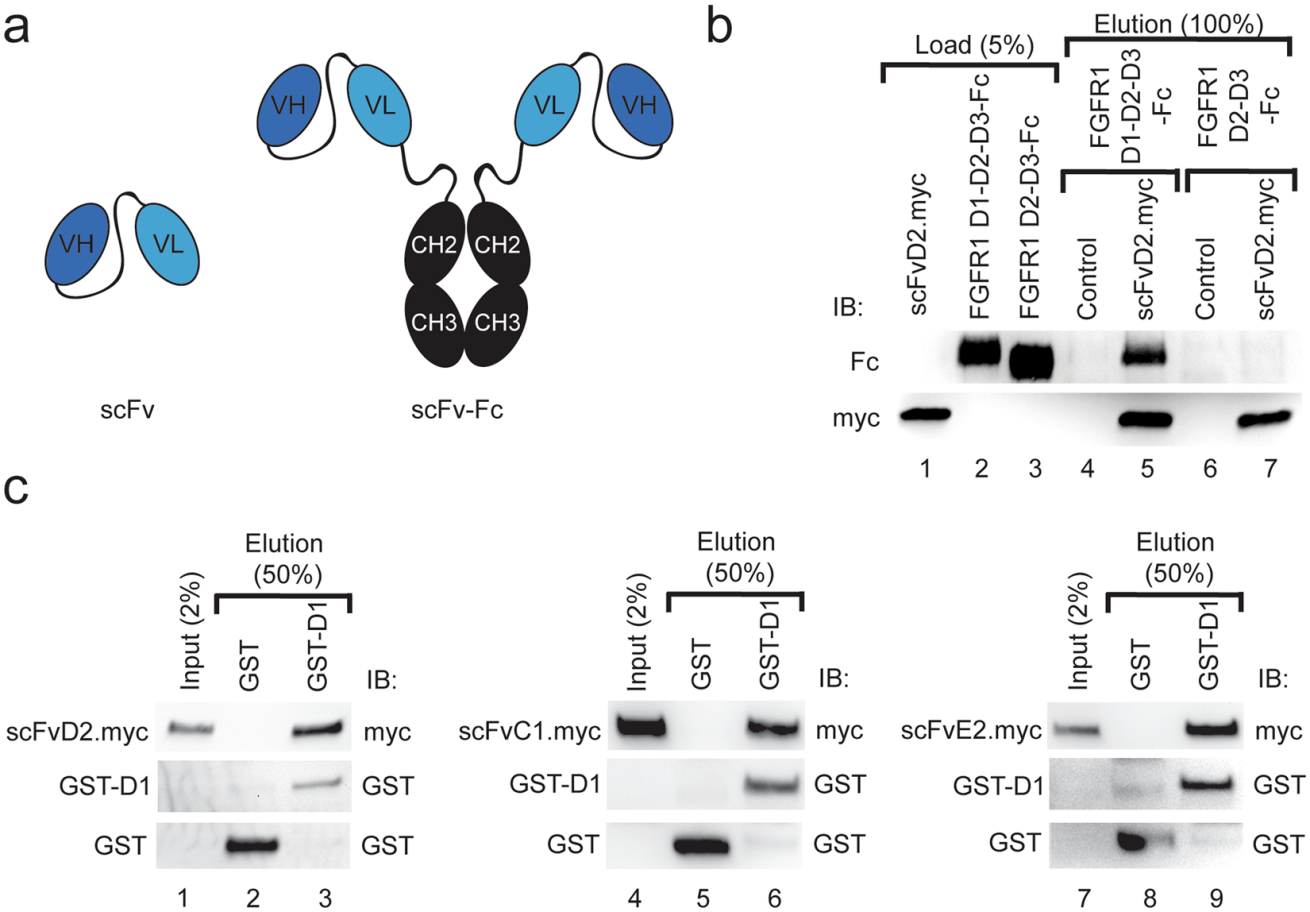
Antibody fragments bind to the domain D1 of FGFR1. (**a**) Schematic representation of anti-FGFR1 antibody fragments used in this study. The scFv antibody format contains single antigen binding site formed by variable domains of heavy and light antibody chains (VH and VL), whereas scFv-Fc antibody fragments contain two identical binding sites for antigen fused by constant domains of heavy chain of human IgG1 (CH2 and CH3). (**b**) Analyzed antibodies recognize epitopes within domain D1 of FGFR1. scFvD2.myc was bound to the anti-c-Myc agarose and incubated with either purified full length extracellular part of FGFR1 fused with Fc fragment (FGFR1 D1-D2-D3-Fc) or with the Fc-fusion of the extracellular part of FGFR1 lacking domain D1 (FGFR1 D2-D3-Fc). Proteins bound to scFvD2.myc were analyzed with anti-Fc antibodies. (**c**) Direct Interaction of scFv’s with the domain D1 of the FGFR1. Recombinant GST (Control) and GST-tagged domain D1 of the FGFR1 (GST-D1) were bound to Glutathione Sepharose and incubated with scFv proteins. Proteins bound to GST and GST-D1 were eluted and analyzed by Western blotting using specific antibodies. Cropped blots were displayed, full size blots are included in Supplementary Information.

A panel of FGFR1-specific scFv antibody fragments was generated by phage display screening of Tomlinson I and J libraries: scFvD2, scFvC1 and scFvE2. To obtain bivalent antibody fragments selected scFv proteins were fused with the Fc region of human IgG, resulting in scFvD2-Fc, scFvC1-Fc and scFvE2-Fc (Fig. 1a). Generated antibody fragments in both studied formats bind with high affinity to FGFR1 *in vitro* and can interact with full length FGFR1 produced by the model U2OSR1 cells (Fig. S1A and B, Fig. S2, Fig. S4). We screened the binding sites of antibody fragments on FGFR1 with pull down assays using full length extracellular part of FGFR1 (composed of three domains D1-D2-D3) and receptor truncated form lacking the N-terminal D1 domain (D2-D3). All selected antibody fragments interacted with full length extracellular part of FGFR1 and these interactions were fully abolished in the absence of D1 domain (Fig. 1b, Fig. S3). Next, using recombinant D1 domain of FGFR1 we confirmed that antibody fragments bound directly to D1 domain of FGFR1 (Fig. 1c). Since D1 was produced in bacterial system, we found that binding of selected antibody fragments to FGFR1 is independent of receptor glycosylation state.

**Figure 2:**
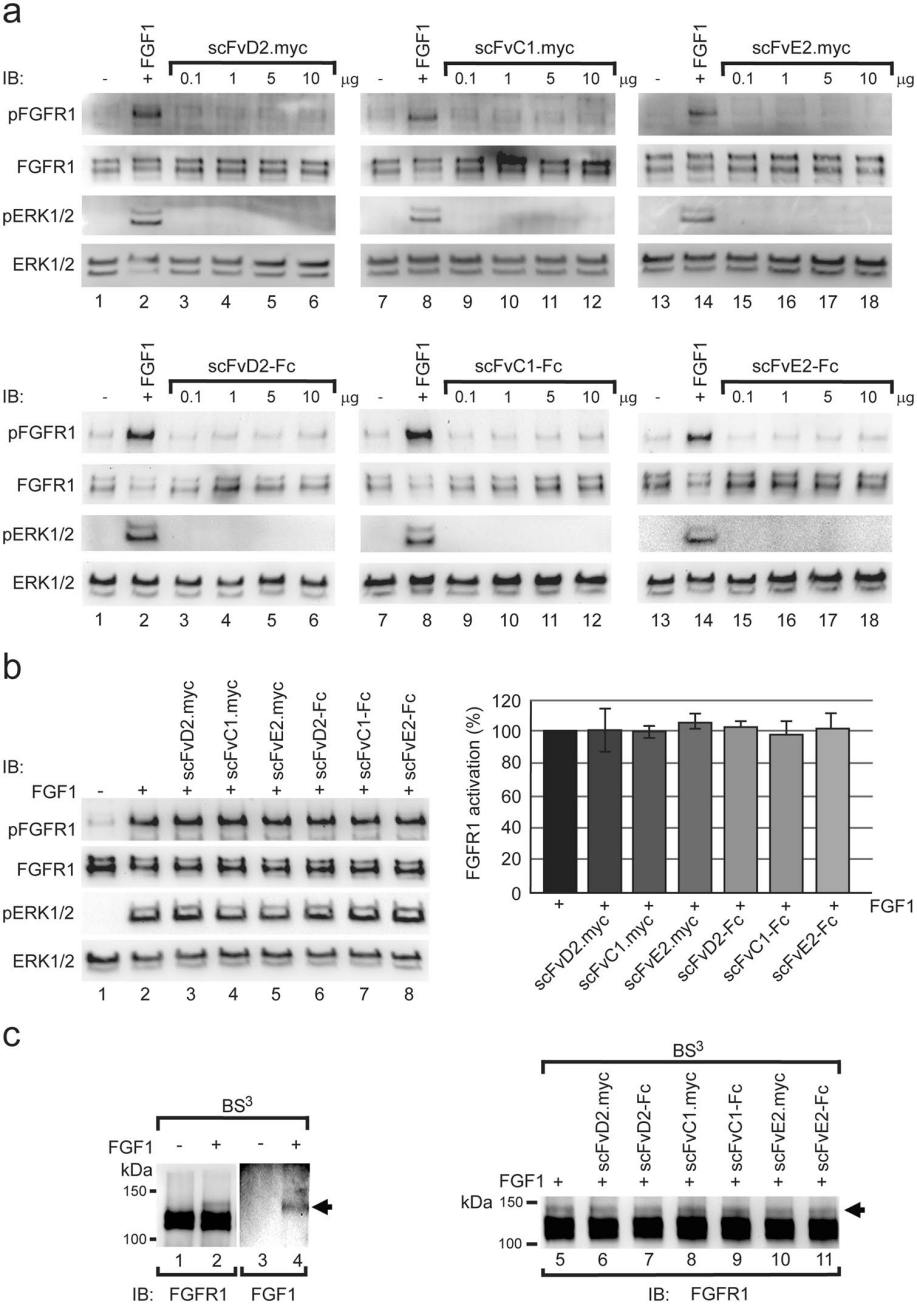
Antibody fragments do not disturb the FGFR1 function. (**a**) Serum starved NIH3T3 cells were stimulated with FGF1 and increasing doses of antibody fragments. The activation of FGFR1 was assessed by Western blotting using antibodies against phosphorylated FGFR1 (pFGFR1), phosphorylated ERK1/2 (pERK1/2), total FGFR1 and total ERK1/2 (loading controls). (**b**) Antibodies in the scFv and scFv-Fc formats have no influence on the FGFR1 activation. Serum starved NIH3T3 cells were stimulated for 15 min with FGF1 alone or in the presence of 10x molar excess of antibody fragments. Cells were lysed and the level of phosphorylated FGFR1 and ERK1/2 (pFGFR1 and pERK1/2) and total level of ERK1/2 and FGFR1 (loading controls) was assessed using specific antibodies and quantified (n = 3, error bars represent SD). (**c**) FGF1-FGFR1 interaction monitored with chemical crosslinking. U2OSR1 cells were incubated with FGF1 and crosslinked with BS^3^. FGF1-FGFR1 crosslinking product was formed that was detected both with anti-FGFR1 and anti-FGF1 antibodies (left panel, arrow). The influence of antibody fragments on the FGF1-FGFR1 interaction was monitored with chemical crosslinking. U2OSR1 cells were incubated with FGF1 in the presence or absence of various antibodies and subjected to crosslinking with BS^3^. Formation of FGF1-FGFR1 complex was monitored by Western blotting using anti-FGFR1 antibodies. Cropped blots were displayed, full size blots are included in Supplementary Information.

FGF1 interacts with D2 and D3 domains of FGFR1, which leads to receptor transphosphorylation and initiation of downstream signaling cascades^36^. We tested whether antibody fragments can, as a consequence of interaction with D1 of FGFR1, activate the receptor. Addition of FGF1 induces FGFR1 autophosphorylation and phosphorylation of downstream effector kinase ERK1/2 (Fig. 2a). Interestingly, antibody fragments in both scFv and Fc format were not able to activate FGFR1, as the phosphorylation status of FGFR1 and ERK1/2 was not altered (Fig. 2a). Next, to study whether FGFR1-specific antibody fragments can influence FGFR1 activity, we incubated serum starved NIH3T3 cells with FGF1 alone or in the presence of the excessive amounts of antibody fragments and monitored FGFR1 activation by Western blotting. Interestingly, none of the studied antibodies blocked FGF1-dependent activation of FGFR1, as detected by ERK1/2 and FGFR1 specific phosphorylation (Fig. 2b). Moreover, we analyzed whether obtained antibody fragments can influence FGF1-FGFR1 interaction with chemical crosslinking. Crosslinking of U2OSR1 cells with membrane-impermeable crosslinker BS^3^ in the presence of FGF1 allowed for visualization of FGF1-FGFR1 complex, as a distinct high molecular weight crosslinking product, containing both FGF1 and FGFR1 was detected (Fig. 2c, left panel). Formation of the FGF1-FGFR1 crosslinking product was not blocked by the presence of excessive amounts of antibody fragments in scFv as well as in scFv-Fc format (Fig. 2c, right panel). We confirmed that antibody fragments and FGF1 bind to distinct epitopes on FGFR1 by affinity analysis using SPR (Fig. S4), and by the isolation from FGFR1 positive cells ternary complexes, containing FGF1, FGFR1 and antibody fragments (Fig. S5). Interestingly, binding of all studied antibody fragments inhibits interaction of FGFR1 with its co-receptor β-Klotho as determined by BLI technique (Fig. S6). This result implies that binding of scFv proteins to D1 domain provides a steric barrier for interaction of the FGFR1 with β-Klotho.

All these data show that tested antibody fragments recognize epitopes within D1 domain of FGFR1 and do not disturb ligand recognition and receptor functions.

### Bivalency of the antibody fragments is required for their efficient internalization

**Figure 3:**
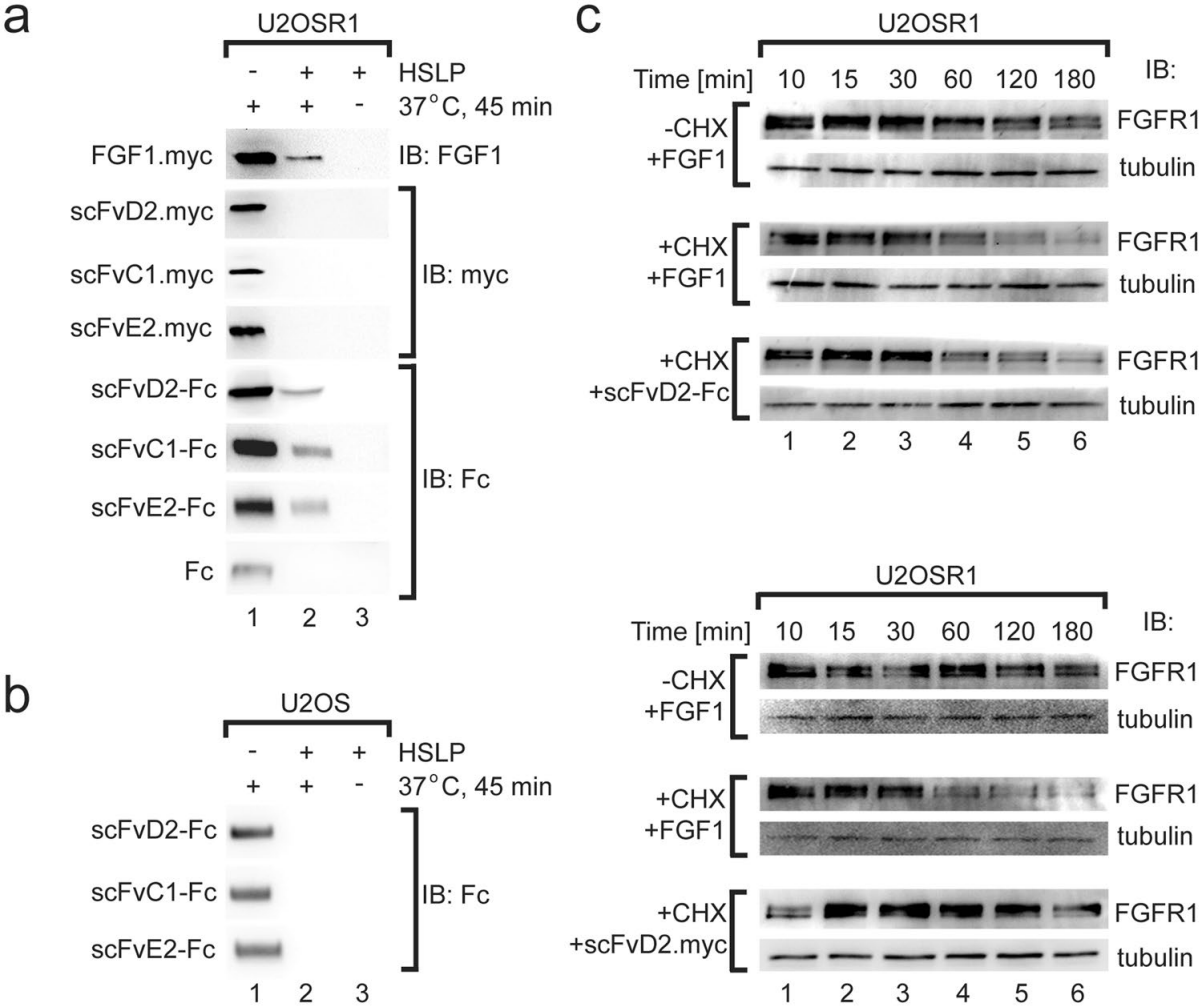
Bivalent scFv-Fc antibodies are internalized into cells in the FGFR1-dependent manner. (**a**) Antibodies in the bivalent scFv-Fc format are internalized into cells that overproduce FGFR1. Serum starved U2OSR1 cells, that overproduce FGFR1 were incubated with FGF1.myc, scFv.myc proteins or scFv-Fc antibody fragments to allow for formation of antibody-FGFR1 and FGF1.myc-FGFR1 complexes. Cells were then shifted for 45 min to 37 °C to initiate internalization. The internalization reaction was stopped by cooling down the cells on ice. The surface bound FGF1.myc or antibody fragments were removed by washing with low pH buffer containing high salt concentration (HSLP) and internalized FGF1.myc, scFv.myc and scFv-Fc proteins were recovered from cell lysates with anti-c-Myc (for FGF1.myc and scFv proteins) and Protein A Sepharose (for scFv-Fc proteins). Internalized proteins were detected by Western blotting using specific antibodies. (**b**) Internalization of scFv-Fc antibody fragments is strictly dependent on the level of FGFR1. Control U2OS cells that contain only very low level of FGFR1 were not able to internalize scFv-Fc proteins. (**c**) FGF1 and bivalent scFv-Fc antibody fragments stimulate FGFR1 degradation. Serum starved U2OSR1 cells were incubated with FGF1, scFvD2 or scFvD2-Fc in the presence or absence of cycloheximide (CHX). Cells were lysed and the level of FGFR1 was assessed by Western blotting. Tubulin was used as a loading control. Cropped blots were displayed, full size blots are included in Supplementary Information.

Next, we studied the internalization of selected antibody fragments. To analyze the dependence of antibody fragment internalization on FGFR1, we employed cell line overproducing FGFR1 (U2OSR1) and appropriate control cells (U2OS) (Fig. S2). First, we validated our experimental setup using natural ligand of FGFR1, FGF1 that upon receptor binding evokes its dimerization and subsequent activation. Receptor activation is followed by the internalization of FGF1-FGFR1 complexes into the cells via clathrin and dynamin dependent endocytosis^8^. We incubated purified FGF1.myc with serum-starved U2OSR1 cells on ice to allow for formation of ligand-receptor complexes and then cells were shifted to 37 °C for 45 min to induce receptor-mediated endocytosis. The internalization of FGF1-FGFR1 complexes was stopped by placing the cells on ice. The non-endocytosed, cell surface-bound FGF1 was subsequently removed by washing the cells with buffer containing high salt concentration and low pH (HSLP)^9^. Cells were then lysed and internalized FGF1.myc was recovered by immunoprecipitation and detected by Western blotting. We observed that about 3% of added FGF1 was internalized into U2OSR1 cells under these conditions (Fig. 3a). Next, we studied the internalization of FGFR1-specific antibody fragments. All antibodies in the scFv format were able to interact with USOSR1 cells (Fig. 3a, lane 1, Fig. S1A), but formation of scFv-FGFR1 complexes did not lead to their internalization (Fig. 3a, lane 2). In contrast, the same scFv proteins in the bivalent Fc format were efficiently internalized into FGFR1-overproducing cells (Fig. 3a, lane 2). Purified Fc fragment was not taken up by U2OSR1 cells, suggesting that internalization of scFv-Fc proteins is not triggered by the Fc fragment itself, but it rather occurs due to the bivalency of scFv-Fc antibodies (Fig. 3a). Internalization of scFv-Fc antibodies was strictly dependent on FGFR1, as control U2OS cells that do not overproduce FGFR1 (Fig. S2) were not able to internalize scFv-Fc antibodies (Fig. 3b).

For further in detail studies we selected representative antibody in two formats: the monovalent scFvD2.myc and the bivalent scFvD2-Fc. To assess whether internalization of scFv-Fc-FGFR1 complexes is a result of basal recycling of plasma membrane components or an antibody-induced event, we analyzed kinetics of FGFR1 degradation upon inhibition of protein synthesis. First, we incubated U2OSR1 cells with FGF1 in the presence or absence of cycloheximide. In line with the published data, we observed that in the presence of cycloheximide FGF1 induced time-course degradation of FGFR1 (Fig. 3c)^19^. In the absence of cycloheximide FGF1-induced depletion of FGFR1 was largely compensated by the synthesis of novel FGFR1 (Fig. 3c). Similarly to FGF1, also scFvD2-Fc protein induced degradation of FGFR1, but the same antibody in the scFv format was not able to trigger receptor degradation (Fig. 3c). Since FGFR1 is strongly overproduced by the U2OSR1 cells, we also sought to test cell line NIH3T3 with genetically unaltered, moderate expression of FGFR1 (Fig. S2). We confirmed that scFvD2-Fc was internalized by NIH3T3 cells in a similar manner to U2OSR1 cells, and the monovalent scFvD2.myc was not (Fig. S7). These results show that, in contrast to monovalent form, the bivalent Fc format allows for internalization of FGFR1-specific antibody fragments. Moreover, our data demonstrate that antibody fragments in the Fc format trigger endocytosis and subsequent degradation of FGFR1 in the absence of natural receptor ligand.

### The internalization of antibody fragments is independent of FGFR1 activation

**Figure 4:**
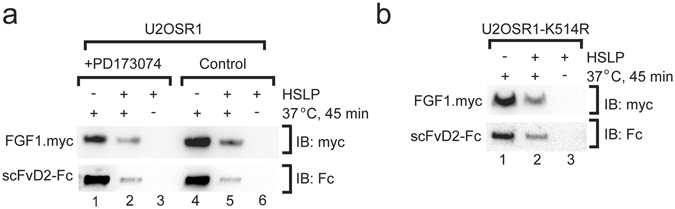
The activation of FGFR1 is not required for the internalization of scFvD2-Fc antibody. (**a**) Internalization of scFvD2-Fc and FGF1 is not affected by the presence of potent FGFR kinase inhibitor PD173074. U2OSR1 cells were preincubated with either DMSO (control) or PD173074 (100 nM) for 15 min and the internalization of FGF1 and scFvD2-Fc was analyzed as in Fig. 3a. (**b**) FGF1 and scFvD2-Fc are taken up by U2OSR1 K514R cells that produce kinase-dead mutant of FGFR1 (as in Fig. 3a). Cropped blots were displayed, full size blots are included in Supplementary Information.

Next, we analyzed whether the internalization of antibody fragments in the Fc format requires FGFR1 activation. To validate that the uptake of scFvD2-Fc occurs without FGFR1 activation, we studied the internalization of FGF1 and scFvD2-Fc under conditions where FGFR1 kinase activity is blocked by a potent inhibitor PD173074. We observed that PD173074 has no influence on the internalization of both FGF1 and scFvD2-Fc (Fig. 4a). To verify our results obtained in the presence of chemical inhibitor, we analyzed the uptake of FGF1 and scFvD2-Fc by cells that stably produce kinase-dead mutant of FGFR1 (U2OSR1-K514R)^37–39^. Both FGF1 and scFvD2 are internalized into these cells (Fig. 4b). These results show that internalization of the bivalent antibody fragment and FGF1 does not require activation of FGFR1.

### The bivalent antibody fragments are internalized via clathrin mediated endocytosis

**Figure 5:**
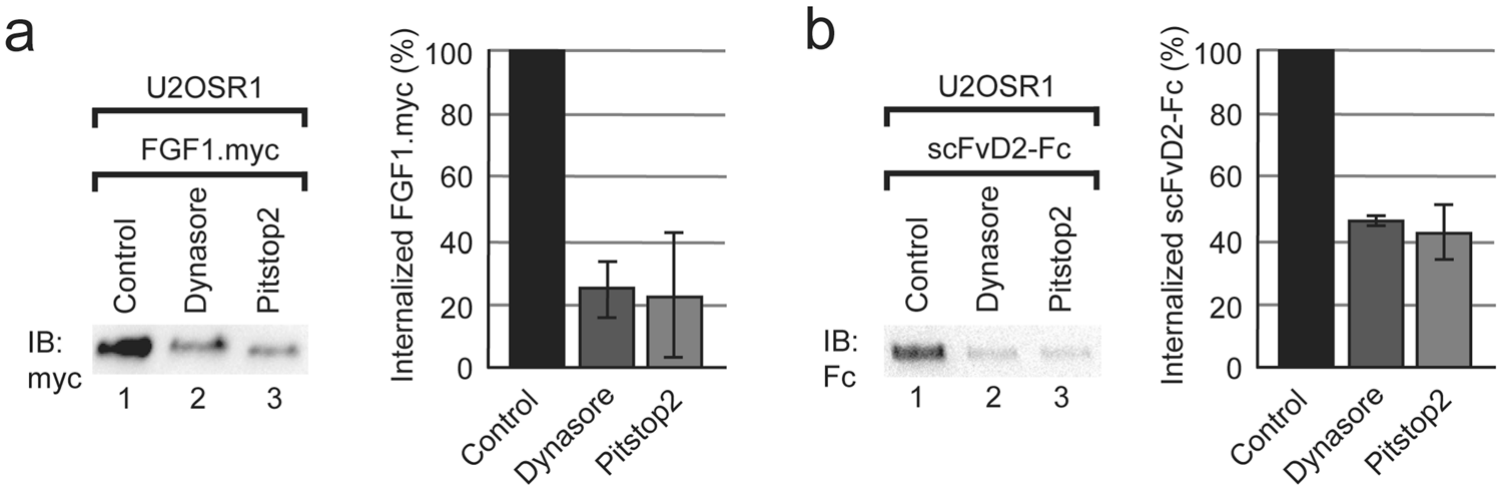
Internalization of FGF1 and scFvD2-Fc occurs via clathrin mediated endocytosis. Internalization of FGF1.myc (**a**) and scFvD2-Fc (**b**) into U2OSR1 cells was studied in the presence of inhibitors of dynamin (Dynasore; 80 µM) and clathrin (Pitstop2; 30 µM). Densitometric quantification of FGF1.myc and scFvD2-Fc internalization was performed with ImageJ from 3 independent experiments. Fraction of internalized FGF1.myc and scFvD2-Fc after treatment with inhibitors is represented as a percentage of untreated control. Error bars represent standard deviation. Cropped blots were displayed, full size blots are included in Supplementary Information.

Endocytosis of FGF1-FGFR1 complexes is mediated by clathrin and dynamin^8^. To study whether scFvD2-Fc utilizes the same molecular machinery as FGF1 for its delivery into the cellular interior, we employed specific inhibitors of clathrin (Pitstop2) and dynamin (Dynasore). We observed that the internalization of scFvD2-Fc, similarly to the internalization of FGF1, was partially blocked by the presence of Pitstop2 and Dynasore (Fig. 5). These data suggest that even though internalization of scFvD2-Fc occurs without FGFR1 activation, it follows the same dynamin/clathrin dependent pathways as FGF1.

### The bivalent antibody fragments trigger FGFR1 dimerization

**Figure 6:**
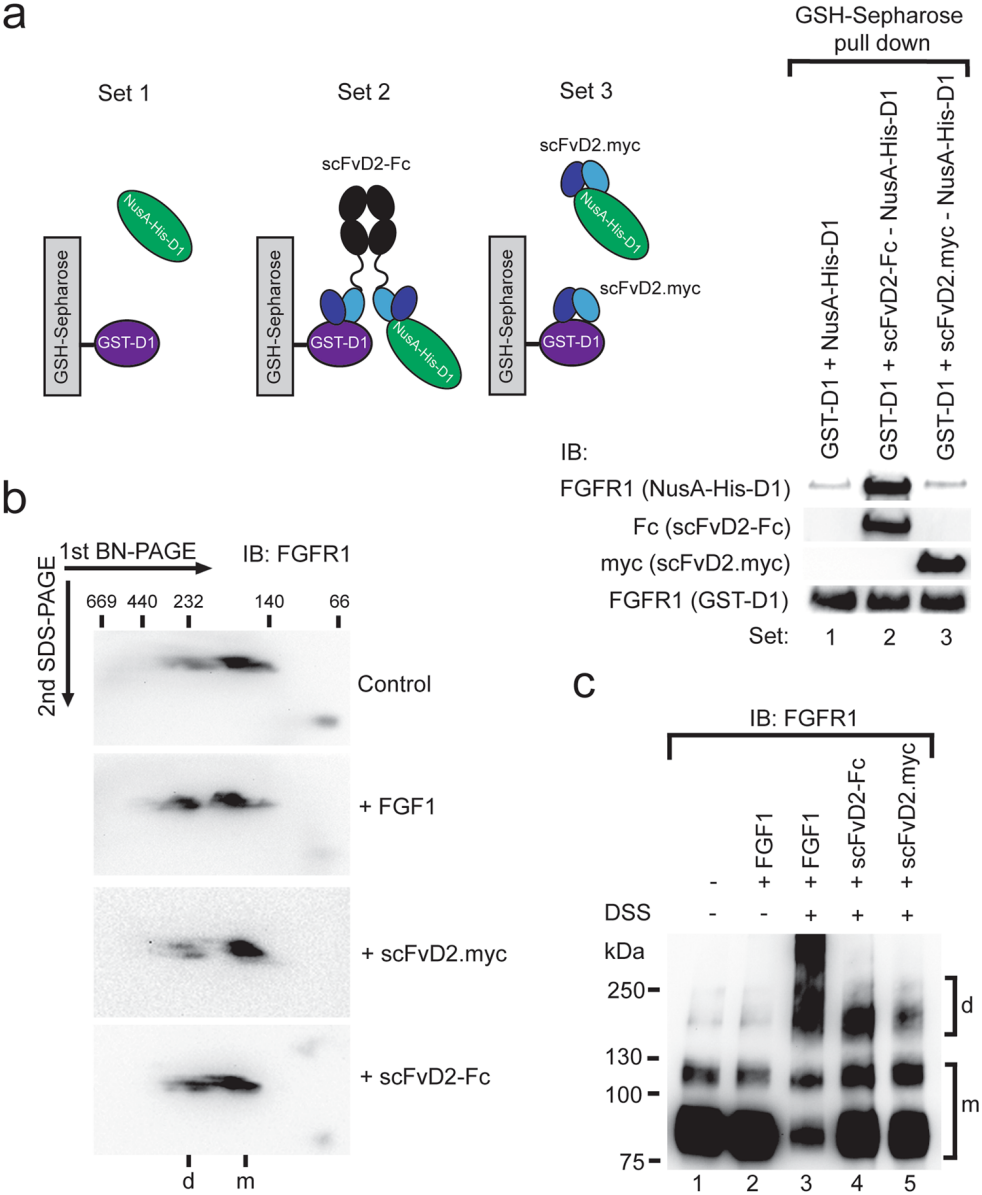
The bivalent scFvD2-Fc antibody fragment induce FGFR1 dimerization. (**a**) The bivalent scFvD2-Fc antibody can simultaneously bind two D1 domains of FGFR1. Purified his-tagged, NusA fusion of domain D1 of FGFR1 (NusA-His-D1) was incubated either alone or in the presence of the bivalent scFvD2-Fc or monovalent scFvD2.myc. Next, preformed antibody fragment-NusA-His-D1 complexes were incubated with purified GST fusion of D1 domain of FGFR1 (GST-D1). Proteins bound to the GST-D1 were analyzed by Western blotting using specific antibodies. The interaction of D1 domains with each other is strongly enhanced only in the presence of the bivalent scFvD2-Fc. (**b**) Oligomeric state of FGFR1 was studied with 2D BN/SDS PAGE. Serum starved NIH3T3 cells were treated with FGF1, scFvD2.myc or scFvD2-Fc. Membrane proteins were solubilized with digitonin and separated in the 1st native dimension using 4–13% BN-PAGE gels. Native gels were sliced and separated in the 2nd denaturing dimension using SDS-PAGE. Oligomeric state of FGFR1 was assessed by Western blotting using FGFR1-specific antibodies. (**c**) Chemical crosslinking reveals that the bivalent scFvD2-Fc antibody fragment can induce FGFR1 dimerization in cells. Serum starved NIH3T3 cells were incubated with FGF1, scFvD2.myc or scFvD2-Fc and crosslinked with DSS. Cells were lysed, FGFR1 was isolated by co-immunoprecipitation and oligomerization of FGFR1 was assessed by Western blotting using receptor-specific antibodies. Addition of FGF1 strongly induces FGFR1 dimerization. The bivalent scFvD2-Fc also induces FGFR1 dimerization, but to the lower extent than FGF1. In contrast the monovalent scFvD2.myc has no impact on the oligomeric state of FGFR1.

It is well established that ligand binding to the FGFR1 triggers receptor dimerization and subsequent activation. Since internalizing scFv-Fc antibodies do not activate FGFR1, we verified whether these antibody fragments are able to induce FGFR1 dimerization. As antibody fragments recognize D1 domain of FGFR1, we analyzed if scFvD2-Fc can simultaneously bind two D1 domains of FGFR1 *in vitro*. We incubated recombinant NusA-His-D1 with scFvD2.myc or with the bivalent scFvD2-Fc and then with recombinant GST-D1 bound to GSH-Sepharose (Fig. 6a). In the control sample (without antibodies) we observed very weak interaction of NusA-His-D1 with GST-D1. The presence of the bivalent scFvD2-Fc strongly enhanced the signal from NusA-His-D1, indicating heterocomplex formation of two D1 domains via scFvD2-Fc, which was not observed for monovalent scFvD2.myc (Fig. 6a). Next, we studied if scFvD2-Fc can induce dimerization of FGFR1 in cells using 2D BN/SDS-PAGE. After incubation of antibody fragments or FGF1 with serum-starved NIH3T3 cells plasma membrane protein complexes were solubilized with mild detergent, digitonin, and then subjected to BN-PAGE. BN-PAGE was followed by 2nd dimension electrophoresis under denaturing conditions and Western blotting. We observed that FGFR1 was present in at least two distinct complexes: majority of FGFR1 is detected in low molecular weight complex, corresponding to the FGFR1 monomer, and little portion of FGFR1 is present in higher molecular weight complexes that likely represent FGFR1 dimers (Fig. 6b). This is in agreement with recently published data pointing on the presence of FGFR1 dimers in the absence of ligands^40, 41^. Addition of FGF1 led to the redistribution of FGFR1 from the monomeric to the dimeric receptor fraction (Fig. 6b). The monovalent scFvD2.myc had no influence on the oligomeric state of the FGFR1, while the bivalent scFvD2-Fc induced FGFR1 dimerization, but to the lower extent than FGF1 (Fig. 6b). We confirmed these results using chemical crosslinking. NIH3T3 cells were incubated with FGF1, scFvD2.myc or scFvD2-Fc and subjected to crosslinking with DSS followed by FGFR1 isolation by immunoprecipitation. The presence of FGF1 led to the efficient formation of high molecular weight species of FGFR1, which seemed to correspond to the FGFR1 dimers. The bivalent scFvD2-Fc also induced FGFR1 dimerization, but to the lower extent than FGF1. In contrast, the monovalent scFvD2.myc was not able to evoke the oligomeric state of FGFR1 (Fig. 6c).

## Discussion

**Figure 7:**
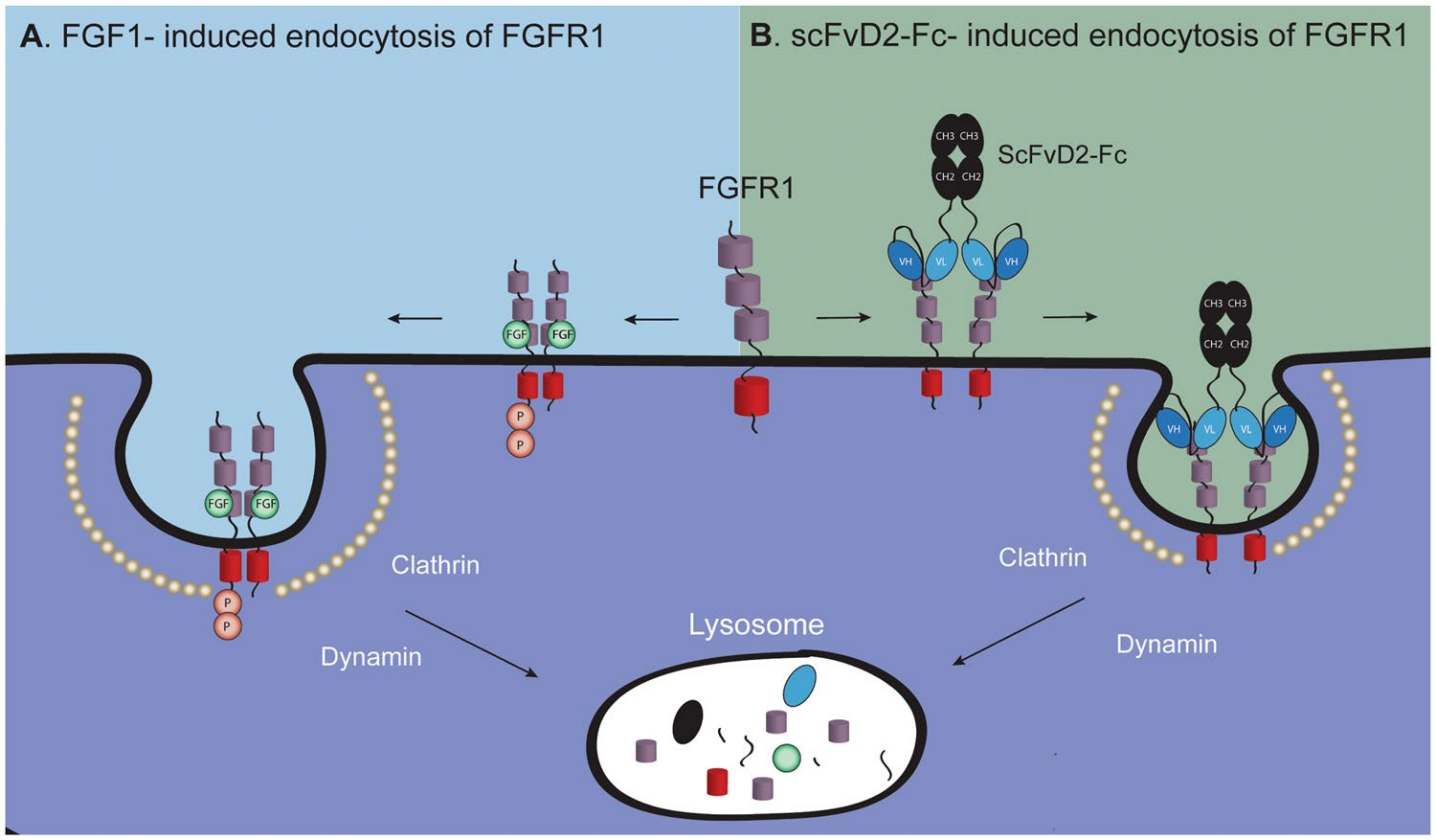
The model of the growth factor- and bivalent antibody-induced endocytosis of FGFR1. (**a**) FGF1 binds to the D2 and D3 domains of FGFR1 and induces FGFR1 dimerization. The conformational change within FGFR1 in the receptor dimer leads to the activation of FGFR1 kinase domain that results in receptor activation by transphosphorylation. Activated FGFR1 recruits intracellular signaling proteins and this results in the initiation of downstream signaling cascades. To downregulate the FGFR1-dependent signals FGFR1 dimer is sequestered into clathrin-coated vesicles and internalized into the cells via CME that requires dynamin. Subsequently FGFR1-containing endosomes are delivered for the lysosomal degradation. (**b**) The bivalent scFvD2-Fc antibody interacts with the D1 domain of FGFR1, leading to receptor dimerization. However, the conformation of the FGFR1 dimer is distinct from the one induced by FGF1, as there is no receptor activation. FGFR1 dimerization likely constitutes the signal for internalization of antibody-receptor complexes, as only bivalent antibody fragments are internalized. Next steps of scFvD2-Fc internalization, similarly to FGF1, employs FGFR1-mediated, clathrin and dynamin-dependent endocytosis. The scFvD2-Fc-FGFR1 complexes are finally degraded, likely in the lysosomes.

Binding of FGF1 to the FGFR1 triggers conformational changes within FGFR1 that lead to the receptor dimerization and concomitant transphosphorylation^1^. Phosphorylated kinase domain of FGFR1 recruits signaling molecules, which further propagate the signal inside the cell. As a consequence of ligand binding activated receptor is sequestered into clathrin coated pits and internalized via CME that requires dynamin^8^. Finally, growth factor-receptor complexes are delivered to the lysosomes for the degradation or recycled to the plasma membrane (Fig. 7a). Although the bivalent anti-FGFR1 antibody fragment utilizes the same endocytic machinery as FGF1, the initial steps of internalization seem to differ in the case of the scFvD2-Fc and FGF1. The bivalent scFvD2-Fc, similarly to FGF1, triggers receptor dimerization. However, it is likely that scFvD2-Fc imposes different conformation or orientation of FGFR1 molecules within receptor dimer that does not allow for FGFR1 transphophorylation, but still permits internalization (Fig. 7b). This may be achieved as antibody fragments bind to the different region of FGFR1 than growth factor (D1 vs D2-D3, respectively). In light of our data we suggest that not the receptor activation, but rather the proximity of two FGFR1 molecules constitutes the signal for FGFR1-mediated endocytosis. Similar findings were recently reported for the other RTK, EGFR, where two receptor molecules have to be brought together to initiate receptor mediated endocytosis^23–25^. It is likely that the endocytic adaptor molecules, which select proteins that have to be internalized can simultaneously bind multiple receptors, thus sensing oligomerized cargo. To support this model, the oligomerization of distinct plasma membrane proteins by combination of streptavidin and biotinylated ligands enhanced their internalization^42^. Moreover, it was shown that crosslinking of various antibodies increased their internalization rates^29, 43, 44^. Our hypothesis is further supported by the observation that endocytosis of the scFvD2-Fc and FGF1 occurs efficiently when FGFR1 kinase activity is blocked either by chemical inhibitor or specific mutation in kinase domain. It was reported that the internalization of FGF1-FGFR1 complexes and subsequent translocation of the growth factor to the cytosol and nucleus may occur independently of FGFR activation^22^. However, the oligomeric state of FGFR1 in the presence of the FGF1 and kinase inhibitors was not assessed in these studies^22^. Here, using specific anti-FGFR1 antibody fragments that differ only in the valency (monovalent scFvs vs bivalent scFv-Fcs), we have unequivocally demonstrated that the bivalency of the antibody fragments is required for their endocytosis mediated by FGFR1 and that this process does not require receptor kinase activity.

The malfunction in the RTK-dependent signaling pathways (including FGFR1 dependent signaling cascades) is associated with numerous diseases, including cancer. One of the most promising therapeutic approaches for treatment of RTK-dependent cancer includes the use of monoclonal antibodies that target RTKs^5, 45^. These antibodies can function as a receptor ligand traps, inhibit interaction of ligands with the receptors or block receptor oligomerization. Moreover, antibodies can be utilized as a targeting molecules in the ADC strategy, where they are fused to highly cytotoxic drugs^33^. It is considered to be beneficial in the ADC approach when ADC construct is efficiently delivered into cellular interior. Here, we report that it is possible to engineer highly specific bivalent antibody fragments that target FGFR1, trigger receptor dimerization and efficient internalization without activation of the receptor. Our findings may impact the rational design of therapeutic antibodies for ADC in the treatment of cancer.

## Methods

### Antibodies and reagents

The primary antibodies directed against FGFR1 (#9740), phospho-FGFR1 (pFGFR1; #3476), ERK1/2 (#9102) and phospho-ERK1/2 (pERK1/2; #9101) were from Cell Signaling (Danvers, MA, USA). The primary antibodies: anti-FGF1 (sc-1884), anti-myc (sc-40) anti-FGFR1 (sc-121) and anti-GST antibodies (sc-138) were from Santa Cruz Biotechnology (Dallas, TX, USA). Anti-tubulin primary antibody (#T6557) was from Sigma-Aldrich (St Louis, MO, USA). Anti-human IgG (Fc) antibody coupled to HRP (# 4-10-20) was from KPL (Gaithersburg, MA, USA). The rabbit polyclonal antibodies against extracellular part of FGFR1 (OPA2) were generated by Davids Biotechnologie GmbH (Regensburg, Germany) by immunization of rabbits with purified extracellular portion of FGFR1 fused to the Fc fragment of human IgG. Secondary antibodies were from Jackson Immuno-Research Laboratories (West Grove, PA, USA).

Protein A Sepharose and Glutathione Sepharose resins were from GE Healthcare (Piscataway, NJ, USA). Ni-NTA agarose was from QIAGEN (Hilden, Germany) and anti-c-Myc agarose affinity resin was from Sigma-Aldrich (St Louis, MO, USA).

Chemical crosslinkers: disuccinimidyl suberate (DSS) and bis-sulfosuccinimidyl suberate (BS^3^) were from Pierce Biotechnology (Rockford, IL, USA). Geneticin (G-418) was from BioShop (Burlington, ON, Canada). PD173074, Cycloheximide, Dynasore and Pitstop2 were from Sigma-Aldrich (St Louis, MO, USA). Digitonin was from Serva (Heidelberg, Germany).

### Cells

U2OS cells (human osteosarcoma, ATCC #HTB-96), U2OSR1 and U2OSR1 K514R cells (U2OS cells stably transfected with FGFR1 or FGFR1 K514R, kind gift of Dr. E.M. Haugsten from the Department of Molecular Cell Biology (Institute for Cancer Research, Oslo University Hospital)), were cultivated in Dulbecco’s Modified Eagle’s Medium (Biowest, Nuaille, France) supplemented with 10% fetal bovine serum (Thermo Fisher Scientific, Waltham, MA, USA), antibiotics (100 U/ml penicillin, 100 µg/ml streptomycin). For U2OSR1 cells growth media were additionally supplemented with geneticin (1 mg/ml). NIH3T3 (murine embryonic fibroblasts, ATCC #CRL-1658) were grown in Dulbecco’s Modified Eagle’s Medium (Biowest, Nuaille, France) supplemented with 2% bovine serum (Thermo Fisher Scientific, Waltham, MA, USA) and antibiotics (100 U/ml penicillin, 100 µg/ml streptomycin). Cells were cultivated in 5% CO_2_ atmosphere at 37 °C. Cells were seeded into tissue culture plates one day prior start of the experiments.

### Recombinant proteins

Recombinant FGF1 (Met-Ala-FGF1^22–155^) was produced in *E. coli*, as described previously^46^. The Fc fragment and Fc fusions of the full length extracellular portion of FGFR1 and the extracellular part of FGFR1 lacking N-terminal D1 domain were expressed in CHO cells and purified using Protein A Sepharose^47^. Antibody fragments in the scFv format (scFvD2, scFvC1, scFvE2; containing c-Myc) were expressed in *E. coli* HB2151 and purified using Protein A Sepharose. The scFv proteins in the Fc format (scFv-Fc) were expressed in CHO cells and purified on Protein A Sepharose column. The N-terminal portion of FGFR1, comprising D1 domain and subsequent linker region (residues 22–157 of FGFR1) was cloned into pDEST15 and pETG60A using Gateway Cloning (Thermo Fisher Scientific; Waltham, MA, USA), resulting in plasmids pEXP15-D1link (GST-D1) and pEXP60A-D1link (NusA-His-D1), respectively. GST-D1 and NusA-His-D1 proteins were expressed in *E. coli* BL21 CodonPlus (DE3)-RIL (Agilent Technologies; Santa Clara, CA, USA) and purified with Glutathione Sepharose (GST-D1) and Ni-NTA agarose (NusA-His-D1), according to the standard procedure. Human β-Klotho was from R&D Systems (Minneapolis, MN, USA).

### Chemical crosslinking

For the analysis of FGF1-FGFR1 interaction, serum starved U2OSR1 cells were incubated with FGF1 (200 ng/ml), heparin (20 U/ml) and, where indicated, with scFv and scFv-Fc antibody fragments (10 µg/ml) for 15 min at 37 °C. Cells were subsequently cooled down on ice, washed two times with ice-cold PBS and subjected to chemical crosslinking with BS^3^ (6 mM; 20 min on ice). The crosslinker was quenched with 250 mM glycine and cells were subsequently washed three times with PBS and lysed with Laemmli sample buffer. Samples were analyzed by Western blotting using anti-FGFR1 antibodies (Cell Signaling #9740) and anti-FGF1 antibodies (Santa Cruz Biotechnology sc-1884).

For analysis of the oligomeric state of FGFR1, serum starved NIH3T3 cells were incubated with FGF1 (200 ng/ml), heparin (20 U/ml) and with scFvD2 (90 nM) or scFvD2-Fc (90 nM) for 5 min at 37 °C. Cells were cooled down on ice, washed three times with ice-cold PBS and subjected to crosslinking with DSS (5 mM) for 30 min on ice. Crosslinker was quenched with 0.1 M Tris pH 7.5 (for 30 min on ice). Cells were washed two times with ice-cold TBS and lysed with whole cell lysis buffer (WCL: 50 mM Tris, 150 mM NaCl, 1 mM EDTA, 1% Triton X-100, 1 mM PMSF, pH 8.0). Cellular lysate was clarified by centrifugation (14000 rpm, 10 min, 4 °C) and incubated with anti-FGFR1 antibody (sc-121) (1 h at 4 °C). Lysates were subsequently incubated with Protein A Sepharose (1 h at 4 °C), resin was washed with PBS and bound proteins were eluted with Laemmli sample buffer. Samples were analyzed by Western blotting using anti-FGFR1 antibodies (Cell Signaling #9740).

### Pull-down and co-immunoprecipitations

To study the interaction of scFv and scFv-Fc proteins with FGFR1, U2OSR1 cells were lysed in WCL buffer and incubated with 10 µg of scFv or scFv-Fc antibody fragments bound to anti-c-Myc or Protein A Sepharose, respectively. Resins were extensively washed with washing buffer (WB: 50 mM Tris, 150 mM NaCl, 1 mM EDTA, 0.1% Triton X-100, 1 mM PMSF, pH 8.0) and bound proteins were eluted with Laemmli sample buffer. Proteins were separated with SDS-PAGE and analyzed by Western blotting using anti-c-Myc and anti-FGFR1 antibodies (Cell Signaling #9740).

For detection of the ternary complex: FGF1-FGFR1-scFv-Fc, FGF1 (3.3 µg/ml, heparin 20 U/ml) was incubated with U2OSR1 cells for 15 min on ice. Cells were washed with PBS and lysed in WCL buffer. Lysate was clarified (14 000 rpm, 10 min, 4 °C) and incubated with Protein A Sepharose containing 10 µg of appropriate scFv-Fc protein (2 h, end over end shaking, 4 °C). Resin was washed 4 times with PBS and bound proteins were eluted with Laemmli sample buffer. Proteins were separated with SDS-PAGE and analyzed by Western blotting using following antibodies: anti-human IgG-HRP (Fc), anti-FGFR1 (Cell Signaling #9740) and anti-FGF1.

To study the interaction of antibody fragments with purified extracellular parts of FGFR1 *in vitro*, scFv proteins (10 µg) were bound to anti-c-Myc agarose and incubated with the purified Fc-fusions of the full length extracellular part of FGFR1 (FGFR1 D1-D3-Fc) and extracellular fragment of FGFR1 lacking domain D1 (FGFR1 D2-D3-Fc) (10 µg of each receptor variant). Resins were washed 5 times with WB and bound proteins were eluted with Laemmli sample buffer. Proteins were separated with SDS-PAGE and analyzed by Western blotting using antibodies recognizing c-Myc and Fc fragment of human IgG.

To study the interaction of scFv proteins with purified GST-tagged D1 domain of FGFR1 (GST-D1), purified GST (control, 10 µg) and GST-D1 (10 µg) in washing buffer (WB: 50 mM Tris, 150 mM NaCl, pH 7.5) were bound to Glutathione Sepharose and incubated with scFv proteins diluted in WB (10 µg). Resins were washed extensively with WB and bound proteins were eluted with elution buffer (WB with 20 mM glutathione). Eluates were analyzed by Western blotting using anti-c-Myc antibodies (for scFvs), membranes were subsequently stripped and probed with anti-GST antibodies.

For the analysis of the bivalent properties of scFvD2-Fc, purified GST fusion of the domain D1 of FGFR1 (GST-D1, 20 µg) was bound to the Glutathione Sepharose resin. Purified NusA fusion of FGFR1 containing N-terminal His-tag (NusA-His-D1) was incubated for 1 h at 4 °C alone or in the presence of 90 nM scFvD2 or scFvD2-Fc. The antibody fragment-NusA-His-D1 complexes were then incubated for 1 h at 4 °C with Glutathione Sepharose-bound GST-D1. Beads were subsequently washed 4 times with 50 mM Tris, 150 mM NaCl, 0.05% Triton X-100, pH 7.4 and bound proteins were eluted with Laemmli sample buffer. Proteins, after separation by SDS-PAGE were analyzed by Western blotting using antibodies recognizing c-Myc, Fc fragment of human IgG and extracellular part of FGFR1 (OPA2).

### SPR and BLI affinity measurements

Epitope binning of selected scFvs and FGF1 was performed by SPR analysis using Biacore3000 instrument. scFvC1, scFvD2 and FGF1 at 1 µM were tested in pairwise combinations over a CM5 sensor chip coated with about 650 RU of FGFR1 D1-D3-Fc protein. Measurements were performed in PBS-PN buffer (PBS, 0.005% v/v surfactant P20, 0.02% NaN3; pH 7.2) at a flow rate of 30 μL/min. Dissociation of the analyte from the ligand was monitored for 90 s. Chip surface was regenerated with 10 mM glycine, pH 1.5. Sensograms were analyzed using BIAevaluation 4.1 software.

To analyze the impact of antibody fragments on the interaction of FGFR1 with β-Klotho bio-layer interferometry method was employed using Octet RED K2 system (ForteBio). The extracellular part of FGFR1 (FGFR1 D1-D3-Fc; 10 µg/ml) was chemically immobilized on AR2G biosensors and subsequently interaction of FGFR1 with β-Klotho (11 µg/ml) was analyzed. To study the impact of antibody fragments on FGFR1-β-Klotho interaction, immobilized FGFR1 (10 µg/ml) was incubated first with the excess of scFv proteins (30 µg/ml) and then with β-Klotho (11 µg/ml) in the presence of scFv proteins (30 µg/ml).

### Internalization of antibody fragments

For the biochemical analysis of the internalization of FGF1.myc and antibody fragments, serum starved U2OSR1 and U2OS cells were incubated either with FGF1.myc (100 ng/ml, heparin 20 U/ml) or with antibody fragments (scFv and scFv-Fc; 10 µg/ml) for 20 min on ice to allow for the formation of FGFR1-FGF1 and FGFR1-antibody fragment complexes. Next, the cells were shifted to 37 °C for 45 min to induce endocytosis. The internalization was stopped by cooling down the cells on ice. Next, the cells were detached from tissue culture dishes by trypsinization on cold. The non-internalized, surface-bound FGF1.myc and antibody fragments were subsequently removed by washing the cells twice with ice cold high salt low pH buffer (HSLP: 2 M NaCl, 40 mM NaAc, pH 4.0). Cells were then washed with ice-cold PBS and lysed with WCL buffer. After clarifying spin (14000 rpm, 10 min, 4 °C) analyzed proteins were recovered from lysates by co-immunoprecipitations (anti-c-Myc agarose for FGF1.myc and scFv proteins) or pull-down (Protein A Sepharose for scFv-Fc proteins). In the experiments with appropriate inhibitors, cells were pre-incubated with PD173074 (100 nM), Dynasore (80 µM) and Pitstop2 (30 µM) for 15 min at 37 °C.

### FGFR1 activation and degradation

To study the impact of antibody fragments on the FGF1-dependent activation of FGFR1, serum starved NIH3T3 cells were stimulated with FGF1 (20 ng/ml, heparin 20 U/ml) for 15 min in the presence of 10 fold molar excess of antibody fragments. Blots from 3 independent experiments were quantified using ImageJ (signal of phosphorylated proteins was corrected for signal of total proteins (loading control)). The value for FGF1 control was set to 100%, and average values from 3 experiments were shown. Error bars represent SD. To analyze whether antibody fragments can activate FGFR1, serum starved NIH3T3 cells were incubated for 15 min with increasing concentrations of scFv and scFv-Fc proteins. Cells were lysed in Laemmli buffer and subjected to SDS-PAGE and Western blotting.

For analysis of the kinetics of FGFR1 degradation, serum starved U2OSR1 cells were treated with cycloheximide (10 µg/ml), FGF1 (100 ng/ml, heparin 20 U/ml), scFvD2 (10 µg/ml) or scFvD2-Fc (10 µg/ml) for up to 180 min at 37 °C. At distinct time points cells were lysed in Laemmli buffer and subjected to SDS-PAGE and Western blotting.

### 2D Blue Native/SDS-PAGE

For Blue Native PAGE (BN-PAGE) serum starved NIH3T3 cells were stimulated with FGF1 (300 ng/ml, heparin 30 U/ml) or equimolar concentrations of scFvD2 (10 µg) and scFvD2-Fc (40 µg) for 10 min at 37 °C. Next, the cells were cooled down on ice and washed twice with ice-cold PBS. The cellular membrane proteins were subsequently solubilized in digitonin buffer (20 mM Tris, 0.1 mM EDTA, 50 mM NaCl, 10% glycerol, 1 mM PMSF, 2% digitonin) for 15 min at 4 °C. Solubilized proteins were separated from insolubilized material by centrifugation (14000 rpm, 10 min, 4 °C) and separated on 4–13% gradient BN-PAGE gels^48^. For two-dimensional gel analyses, individual gel lanes were isolated from BN-PAGE gels and incorporated into the stacking part of the SDS-PAGE gel. After electrophoresis under denaturing conditions proteins were transferred onto PVDF membrane and detected with anti-FGFR1 antibodies (Cell Signaling #9740).

## Electronic supplementary material

Supplementary Info
