# Supplementary material for: Antibody-induced dimerization of FGFR1 promotes receptor endocytosis independently of its kinase activity
Source: Sci Rep. 2017 Aug 2;7:7121. doi: 10.1038/s41598-017-07479-z (PMC5540934; doi:10.1038/s41598-017-07479-z)
Supplement: Supplementary file 1 — Supplementary Info [file 41598_2017_7479_MOESM1_ESM.pdf]

Supplemental Information for:

Antibody-induced dimerization of FGFR1 promotes receptor endocytosis independently of its kinase activity

Łukasz Opaliński*, Aleksandra Sokołowska-Wędzina, Martyna Szczepara, Małgorzata Zakrzewska and Jacek Otlewski*

Faculty of Biotechnology, Department of Protein Engineering, University of Wroclaw, Joliot-Curie 14a, 50-383 Wroclaw, Poland

*Correspondence:

lukasz.opalinski@uwr.edu.pl

jacek.otlewski@uwr.edu.pl

Fig. S1. Antibody fragments interact with FGFR1 produced by U2OSR1 cells

A. Interaction of antibody fragments in the scFv format with the FGFR1 produced by U2OSR1 cells. Antibody fragments were bound to anti-c-Myc agarose and incubated with the lysates prepared from U2OSR1 cells that overproduce FGFR1. Proteins that interact with scFvs were eluted and the level of FGFR1 was assessed by Western blotting. B. Interaction of antibody fragments in the scFv-Fc format with the FGFR1 produced by U2OSR1 cells. Antibody fragments were bound to Protein A Sepharose and incubated with the lysates prepared from U2OSR1 cells that overproduce FGFR1. Proteins that interact with scFv-Fc proteins were eluted and the level of FGFR1 was assessed by Western blotting.

Fig. S2. The level of FGFR1 in studied cell lines

Equal amounts of U2OS, U2OSR1 and NIH3T3 cells were lysed and the level of FGFR1 was assessed by Western blotting.

Fig. S3. Antibody fragments bind to D1 domain of FGFR1

scFvD1 and scFvE2 (myc-tagged) were bound to the anti-c-Myc agarose and incubated with either purified full length extracellular part of FGFR1 fused with Fc fragment (FGFR1 D1-D2-D3-Fc) or with the Fc-fusion of the extracellular part of FGFR1 lacking domain D1 (FGFR1 D2-D3-Fc). Proteins bound to scFvE2 and scFvC1 were analyzed with anti-Fc antibodies.

Fig. S4. SPR analysis of the competitive binding of antibody fragments and FGF1 to the extracellular portion of FGFR1

SPR sensograms showing epitope binning analysis of scFvs and FGF1. Monomeric fractions of scFvC1, scFvD2 and FGF1 at 1 μM were tested in pairwise combinations over a CM5 sensor chip coated with about 650 RU of FGFR1-Fc protein.

Fig. S5. Antibody fragments and FGF1 do not compete for the binding to FGFR1 produced by model cells

U2OSR1 cells were incubated with FGF1 to allow for the formation of the growth factor-FGFR1 complexes. Cells were lysed and incubated with Protein A Sepharose-bound scFv-Fc antibody fragments. Proteins bound to scFv-Fcs were eluted and analyzed by Western blotting. Since antibody fragments do not interact with FGF1 (data not shown), detection of FGF1 in the eluates of scFv-Fc pull down suggest the presence of ternary complexes: FGF1-FGFR1-scFv-Fcs and thus distinct binding sited for FGF1 and scFv-Fc proteins on FGFR1.

Fig. S6. Antibody fragments block interaction of FGFR1 with β-Klotho

BLI technique was used to assess the interaction of FGFR1 with β-Klotho co-receptor. Extracellular part of FGFR1 was chemically immobilized on AR2G biosensors, reaction was subsequently quenched and sensors were used for binding analysis either with β-Klotho alone (A), or first saturated with the excess of scFv proteins and then incubated with β-Klotho in the presence of appropriate scFv (B-D).

Fig. S7. scFcD2-Fc is internalized by NIH3T3 cells

Serum starved NIH3T3 cells were incubated with scFvD2.myc or scFvD2-Fc to allow for formation of FGFR1-antibody fragment complexes. Cells were shifted to 37ºC to induce endocytosis. Reaction was stopped by cooling down the cells on ice. Surface-bound, non-internalized antibody fragments were removed by washing the cells with

HSLP buffer. Internalized antibody fragments were recovered with anti-c-Myc agarose and Protein-A-Sepharose.

Fig. S8. Full length blots used in the main figures

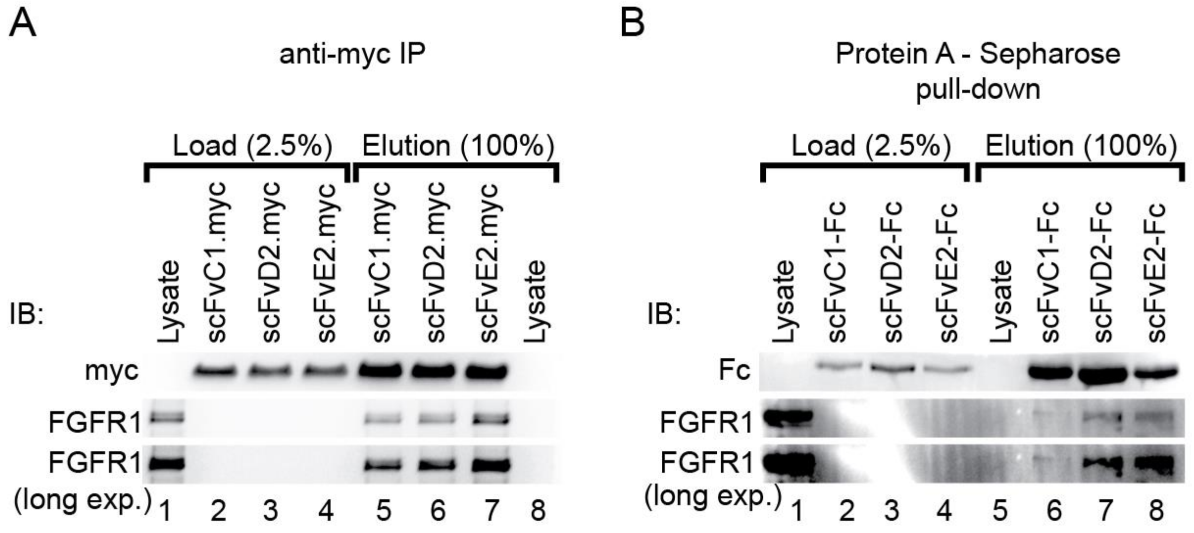

A
anti-myc IP
Load (2.5%)
Elution (100%)
IB:
Lysate
scFvC1.myc
scFvD2.myc
scFvE2.myc
scFvC1.myc
scFvD2.myc
scFvE2.myc
Lysate
myc
FGFR1
FGFR1
(long exp.)
1 2 3 4 5 6 7 8
B
Protein A - Sepharose
pull-down
Load (2.5%)
Elution (100%)
IB:
Lysate
scFvC1-Fc
scFvD2-Fc
scFvE2-Fc
Lysate
scFvC1-Fc
scFvD2-Fc
scFvE2-Fc
Fc
FGFR1
FGFR1
(long exp.)
1 2 3 4 5 6 7 8

Fig. S1

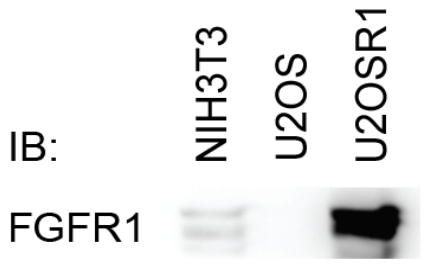

Fig. S2

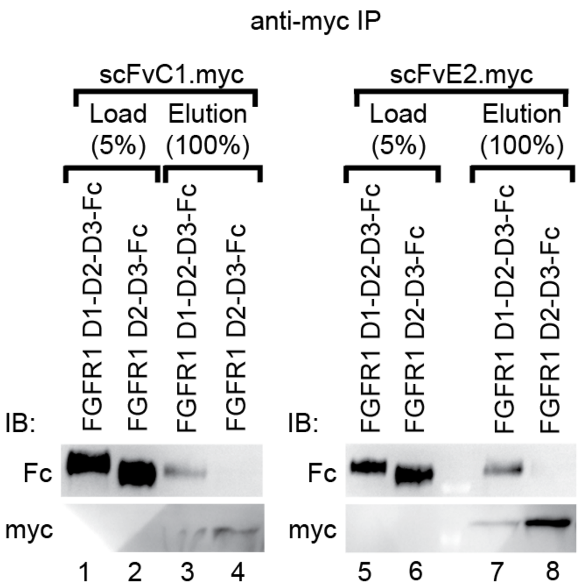

anti-myc IP
scFvC1.myc
scFvE2.myc
Load (5%)
Elution (100%)
Load (5%)
Elution (100%)
FGFR1 D1-D2-D3-Fc
FGFR1 D2-D3-Fc
FGFR1 D1-D2-D3-Fc
FGFR1 D2-D3-Fc
FGFR1 D1-D2-D3-Fc
FGFR1 D2-D3-Fc
FGFR1 D1-D2-D3-Fc
FGFR1 D2-D3-Fc
IB:
Fc
myc
1 2 3 4
IB:
Fc
myc
5 6 7 8

Fig. S3

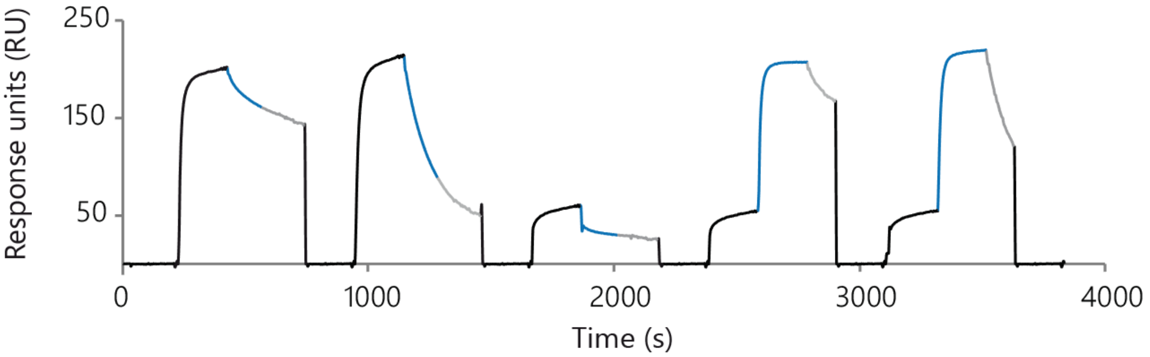

Analyte 1:	scFvD2	scFvC1	FGF1	FGF1	FGF1
Analyte 2:	Buffer	Buffer	Buffer	scFvD2	scFvC1
Dissociation:	Buffer	Buffer	Buffer	Buffer	Buffer

Fig. S4

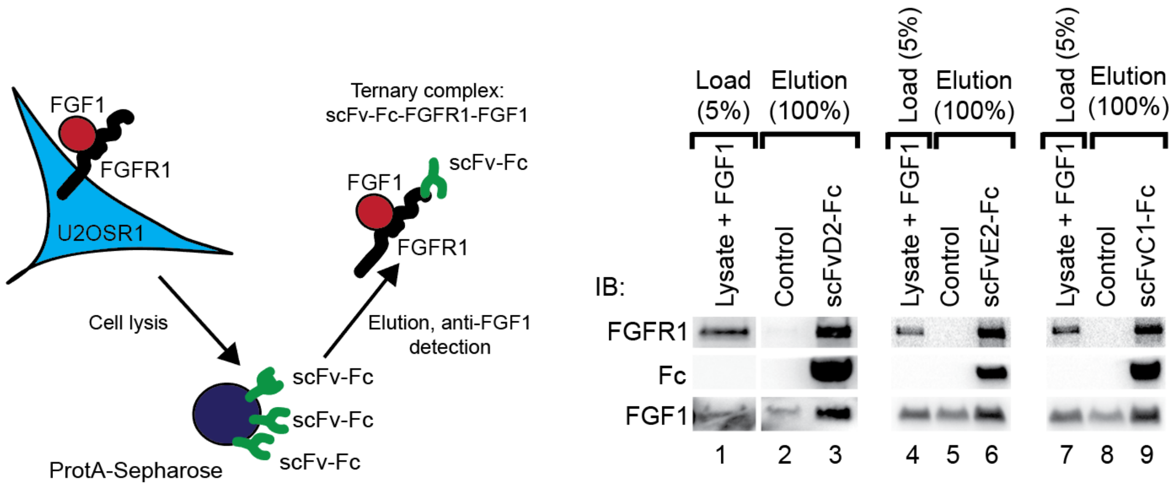

FGF1
FGFR1
U2OSR1
Cell lysis
ProtA-Sepharose
scFv-Fc
scFv-Fc
scFv-Fc
Ternary complex:
scFv-Fc-FGFR1-FGF1
FGF1
scFv-Fc
FGFR1
Elution, anti-FGF1
detection
Load
(5%)
Elution
(100%)
Load (5%)
Elution
(100%)
Load (5%)
Elution
(100%)
IB:
Lysate + FGF1
Control
scFvD2-Fc
Lysate + FGF1
Control
scFvE2-Fc
Lysate + FGF1
Control
scFvC1-Fc
FGFR1
Fc
FGF1
1 2 3 4 5 6 7 8 9

Fig. S5

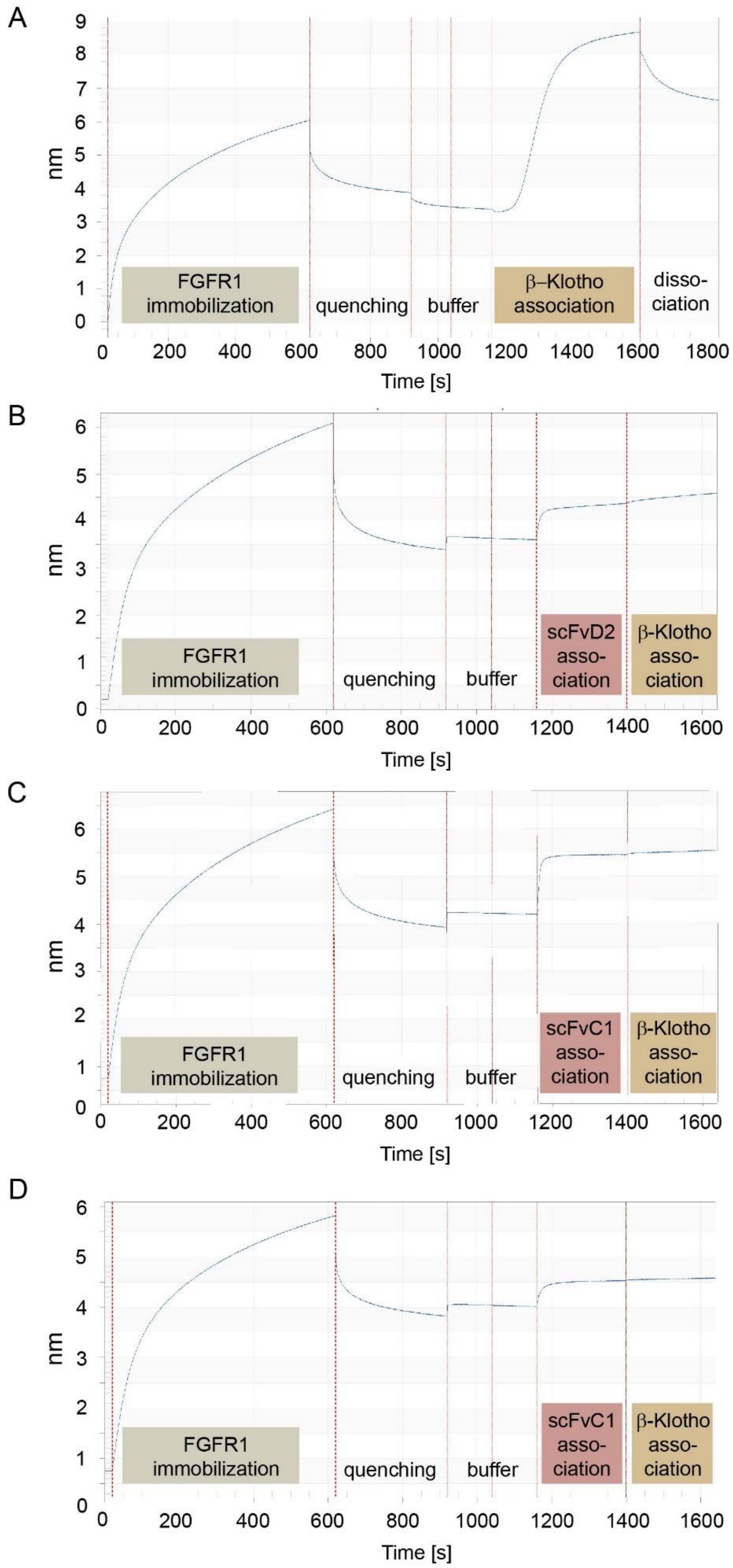
A
B
C
D
nm
Time [s]
FGFR1 immobilization
quenching
buffer
β–Klotho association
disso-ciation
scFvD2 asso-ciation
β-Klotho asso-ciation
scFvC1 asso-ciation

Fig. S6

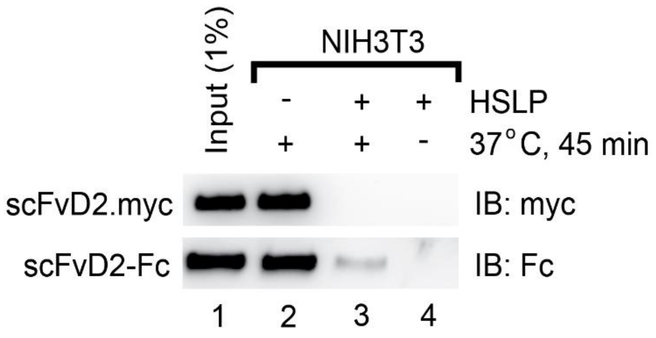
Input (1%)
NIH3T3
- + + HSLP
+ + - 37°C, 45 min
scFvD2.myc
IB: myc
scFvD2-Fc
IB: Fc
1 2 3 4

Fig. S7

Figure 1B

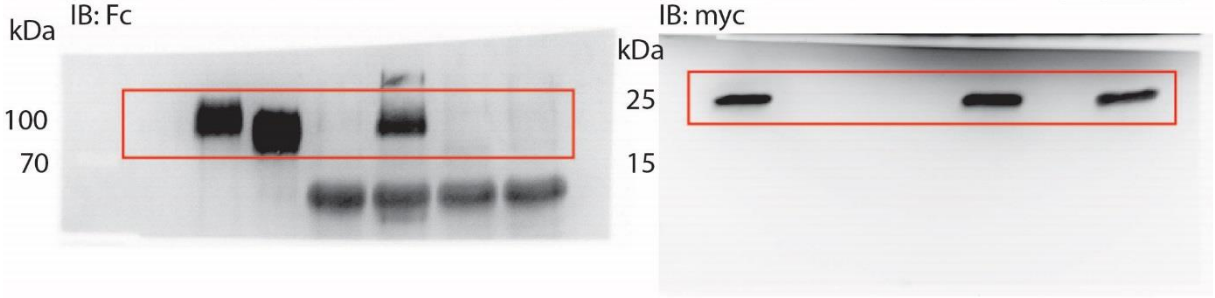

Figure 1C

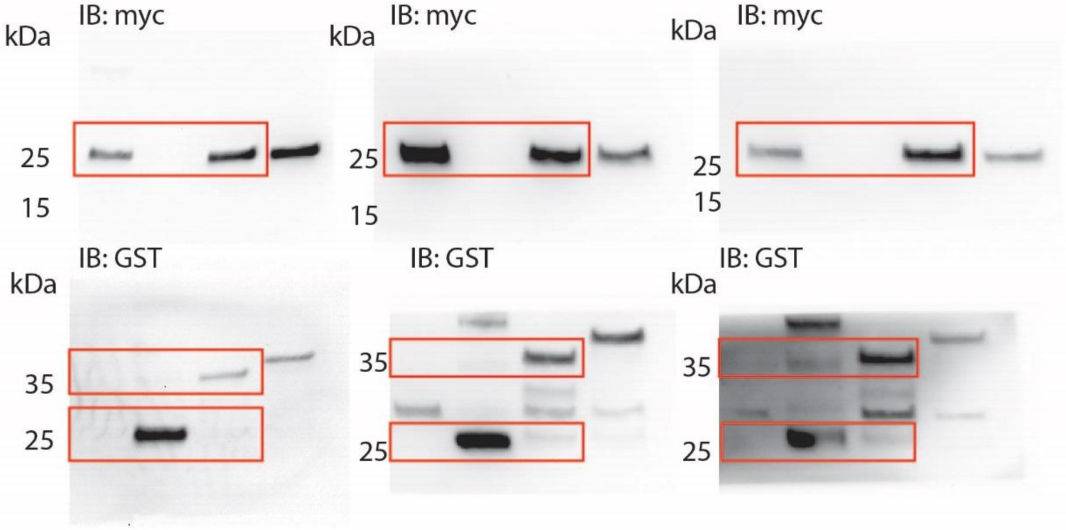

Figure 2A - top panel

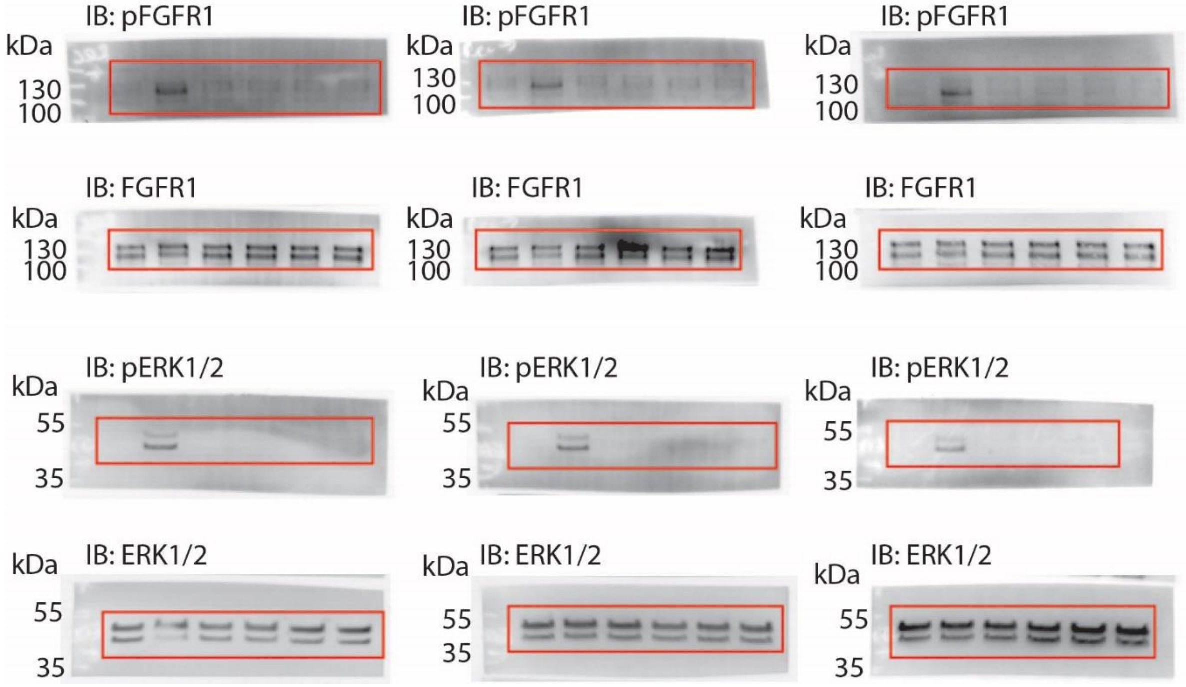

Figure 2A - bottom panel

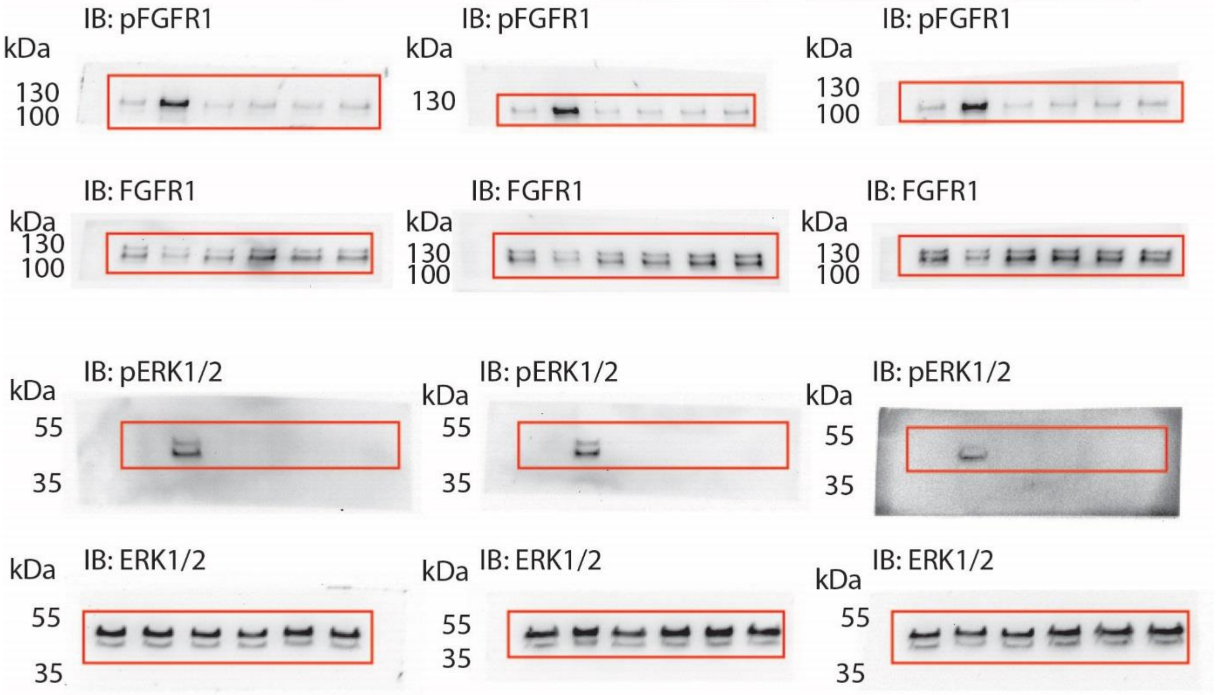

Figure 2B - left panel

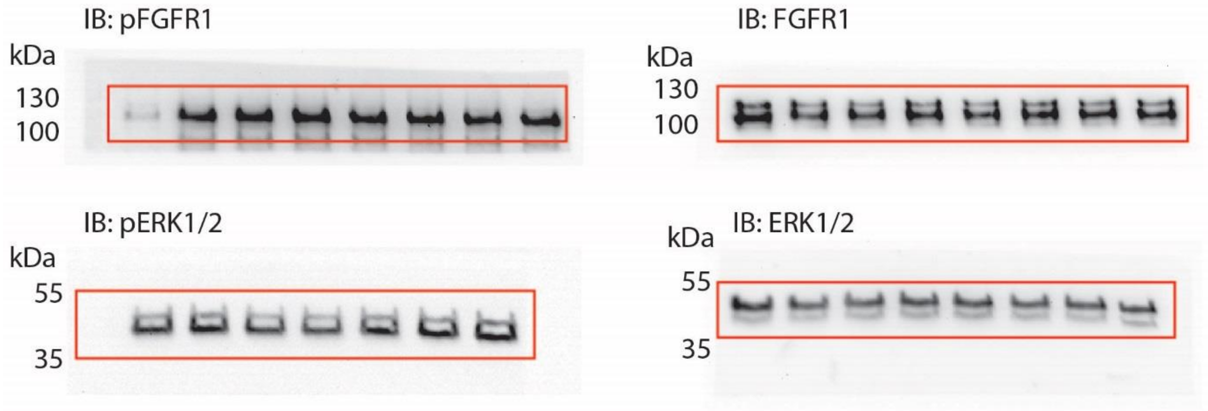

Figure 2C

Figure 2C, left panel

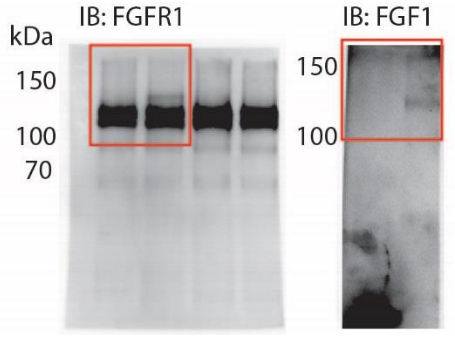

Figure 2C, right panel

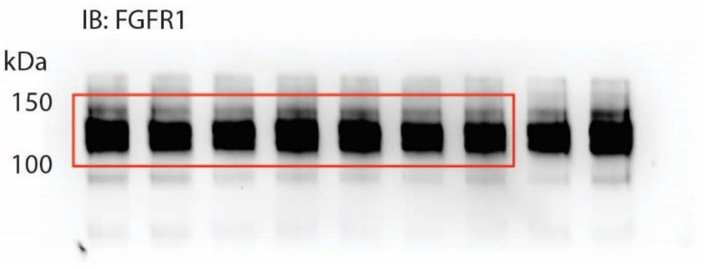

Figure 3A

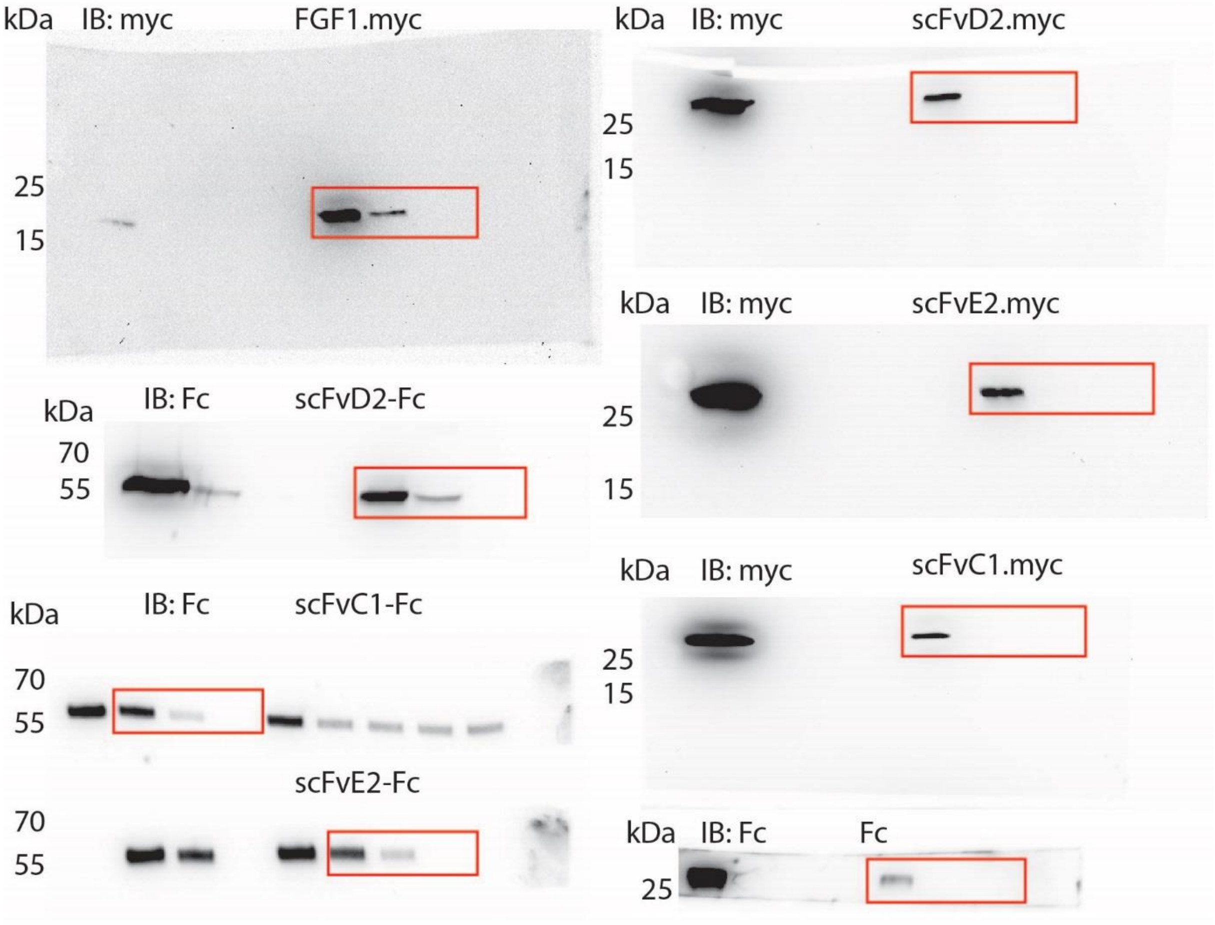

Figure 3B

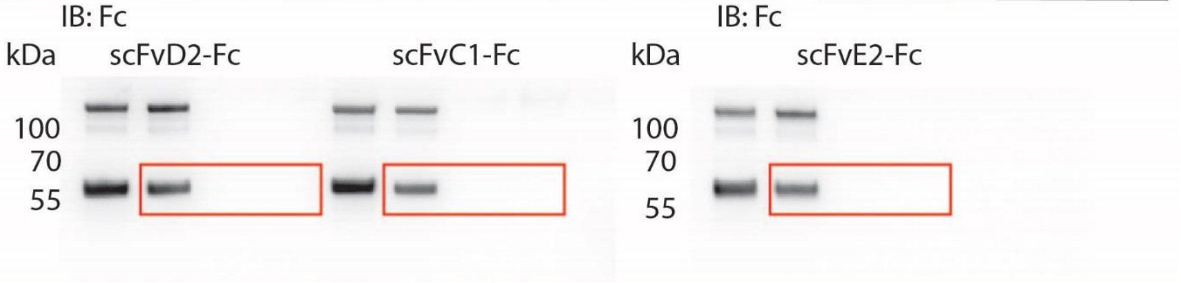

Figure 3C

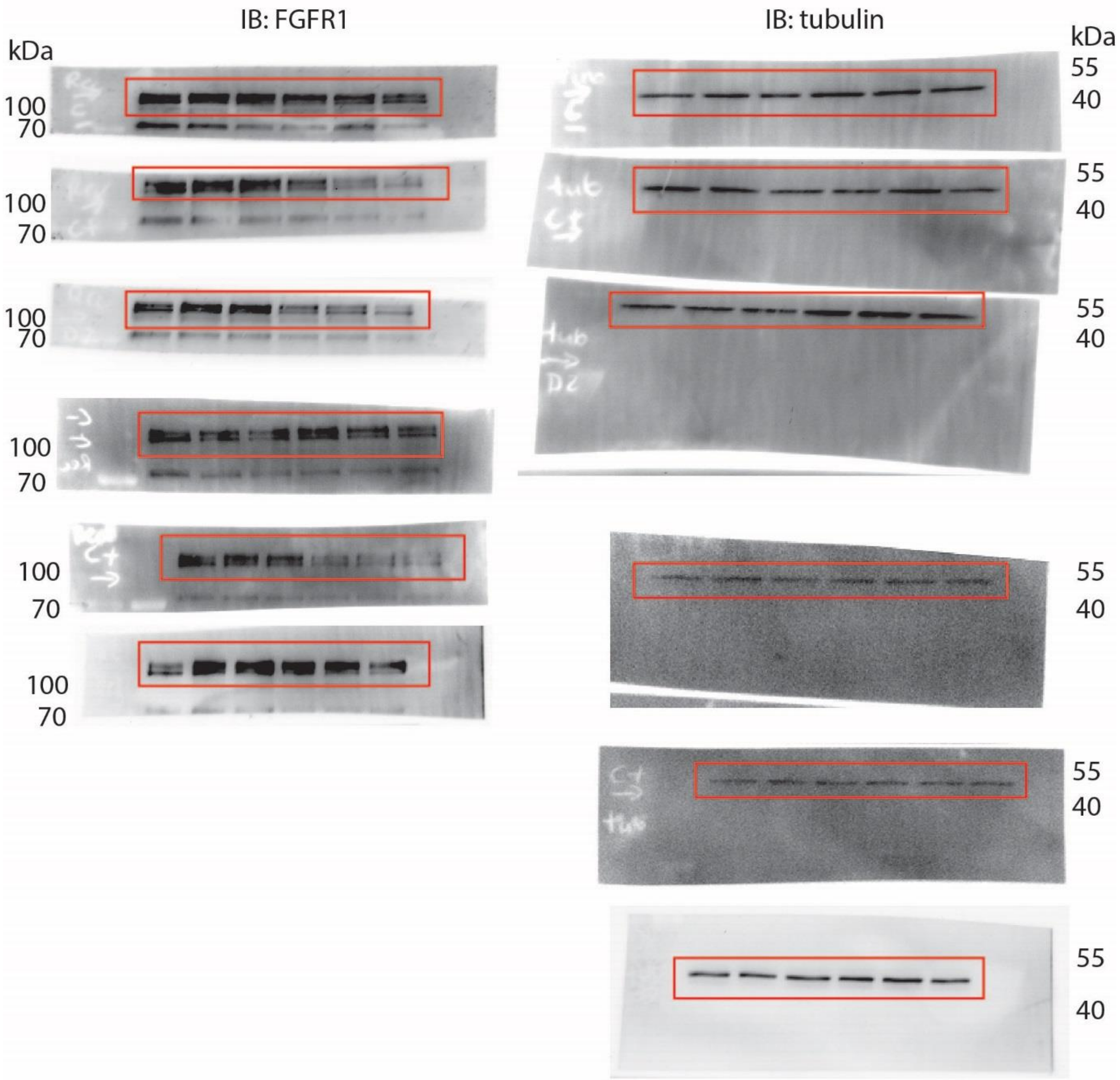

Figure 4A

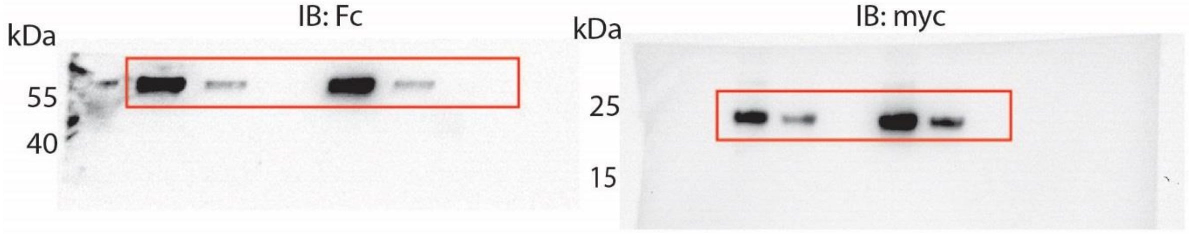

Figure 4B

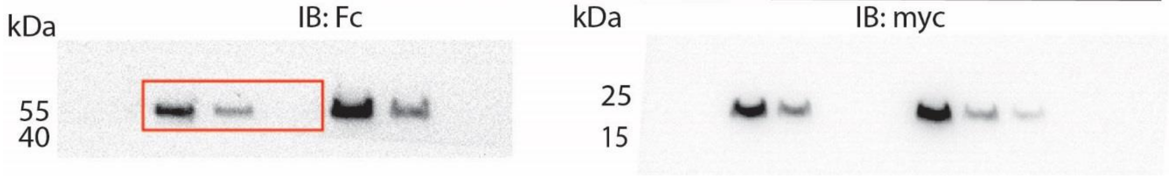

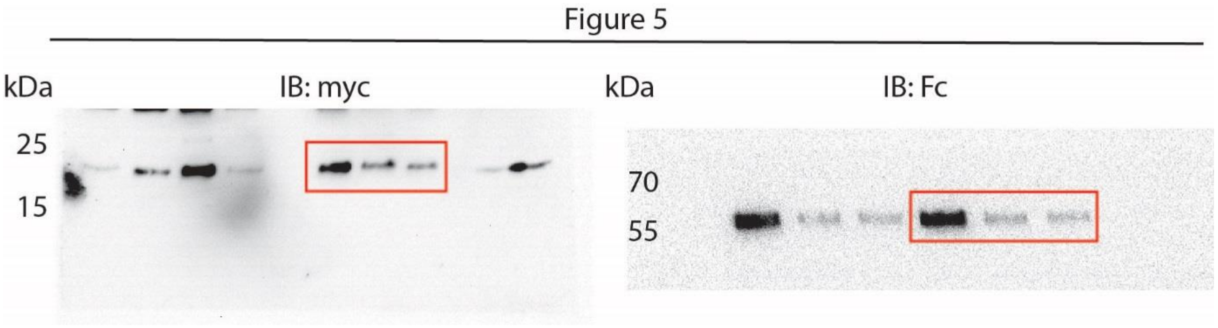

Fig. S8
